# Extranodal Natural Killer/T-cell lymphoma, nasal type: ‘midline lethal granuloma.’ A case report

**DOI:** 10.1186/1746-160X-9-4

**Published:** 2013-01-17

**Authors:** Martha M Tlholoe, Monica Kotu, Razia AG Khammissa, Meschack Bida, Johan Lemmer, Liviu Feller

**Affiliations:** 1 Department of Maxillofacial and Oral Surgery, University of Limpopo, Medunsa Campus, South Africa; 2 Department of Otorhinolaryngology, University of Limpopo, Medunsa Campus, South Africa; 3 Department of Periodontology and Oral Medicine, University of Limpopo, Medunsa Campus, South Africa; 4 Department of Pathology, University of Limpopo, Medunsa Campus, South Africa

**Keywords:** Midline lethal granuloma, Extranodal NK/T cell lymphoma

## Abstract

Extranodal natural killer/T cell lymphoma, nasal type, is a non-Hodgkin lymphoma, most commonly affecting the nasal cavity, paranasal sinuses and nasopharynx. Clinically it is characterised by destruction of facial tissues, commencing in the midline. In most cases it arises from malignant transformation of natural killer cells (NK); sometimes from malignant transformation of cytotoxic T cells.

Extranodal NK/T cell lymphoma, nasal type, is rare, but even more rare in black persons. The purpose of this article is to report a severe case of extranodal NK/T cell lymphoma, nasal type, in an elderly black male.

## Introduction

Extranodal natural killer/T cell (NK/T cell) lymphoma, nasal type, is a rare non-Hodgkin lymphoma originating in the nasal cavity or in the paranasal sinuses. It is strongly associated with Epstein-Barr virus (EBV) infection. Its prevalence is higher in countries in South-East Asia and in Central and South America than in Europe and in North America; it occurs in middle-aged persons and affects males more frequently than females [1-5]. Most cases arise from natural killer cells, only a few from cytotoxic T-cells [6,7].

Clinically, extranodal NK/T cell lymphoma, nasal type, is characterized by progressive midline facial destruction. Initial signs and symptoms include nasal stuffiness, epistaxis and pain, owing to progressive tumour growth in the nose. As the tumour mass enlarges, invading and destroying structures in the upper anterior aerodigestive tract, it becomes progressively necrotic with a purulent discharge. Signs and symptoms are related to the sites involved. Secondary infection and haemorrhage are not infrequent [5,6]. Metastasis is uncommon [8].

As extranodal NK/T cell lymphoma, nasal type, may clinically mimic other destructive disease entities affecting mid-facial structures including other lymphomas, nasopharyngeal squamous cell carcinoma, tertiary syphilis, Wegener granulomatosis and fungal infections, the definitive diagnosis must be based on histopathological, immunological and molecular studies [5].

Localised extranodal NK/T cell lymphoma, nasal type usually responds favourably to radiotherapy. As in any neoplasm the best clinical outcome is achieved when treatment is started early in the course of the disease; but once the tumour has invaded, radiotherapy must be supplemented with chemotherapy. Nevertheless, local recurrence occurs in about 50% of cases. Extensive local invasion, regional lymph node involvement, elevated serum lactate dehydrogenase, raised EBV DNA titres and systemic signs (fever, night sweats, weight loss) are associated with a poor prognosis [3,4,6,9-11], and overall, the prognosis is poor. The five year survival rate is reportedly between 38% and 85%.

About 25% of lymphomas that fulfil the histological, immunological and molecular criteria of diagnosis for extranodal NK/T cell lymphoma, nasal type, may arise in other sites of the upper aerodigestive tract (e.g. nasopharynx, palate), and in sites outside the upper aerodigestive tract including the skin, the gastrointestinal tract and the testis [12]. It appears that no matter where it arises in the upper aerodigestive tract, the course of the disease is similar; but when it arises at other sites it runs a more aggressive clinical course [12,13], frequently disseminating to the spleen, skin or to bone marrow [1,11,14].

To our knowledge, the prevalence of extranodal NK/T cell lymphoma, nasal type, in black persons in sub-Saharan Africa, and in black persons elsewhere in the world is unknown [12]. We report a case of extranodal NK/T cell lymphoma, nasal type, in an elderly black male, that caused extreme destruction and deformity of the midface.

## Case report

**Figure 1:**
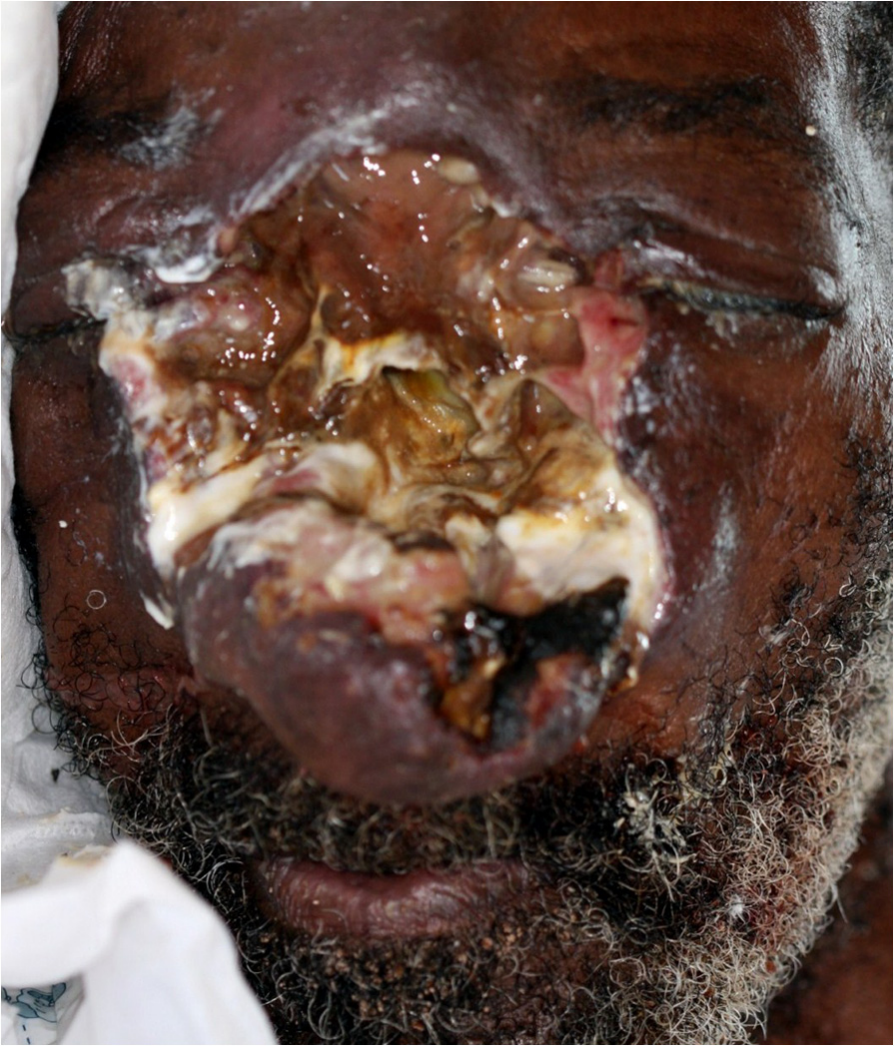
Destruction of the midface with swelling of surrounding facial tissues and lower part of the nose.

A 74-year old black male was referred to the Dr George Mukhari Hospital in Ga Rankuwa, Pretoria with midline facial destruction (Figure 1).

The patient stated that about a year before, a small growth had appeared on his nose, became ulcerated and then enlarged progressively. As the lesion enlarged, his nose became blocked with a discharge, he frequently had headaches, and the sight of his left eye deteriorated. However, owing to his destitute circumstances, it was about a year before he was brought to hospital. He does not smoke or drink alcohol, and had been in good health until the facial condition started.

On admission to hospital he had a fever, headache, conjunctivitis, swelling of the eyelids and of the lower part of the nose, and destruction of the entire midface with indurated swelling of the surrounding tissues (Figure 1). There were no intraoral or extra-facial cutaneous lesions, the regional lymph nodes were not enlarged, and clinically and radiologically the chest was normal.

Intravenous fluids and antibiotics were given and daily irrigation of the wound was started. The patient was not anaemic, was HIV-seronegative and did not have syphilis. There was leucocytosis but not lymphocytosis, and the erythrocyte sedimentation rate, C-reactive protein and lactate dehydrogenase were elevated. Serum IgG was present, but not IgM to EBV cuspid antigen.

**Figure 2:**
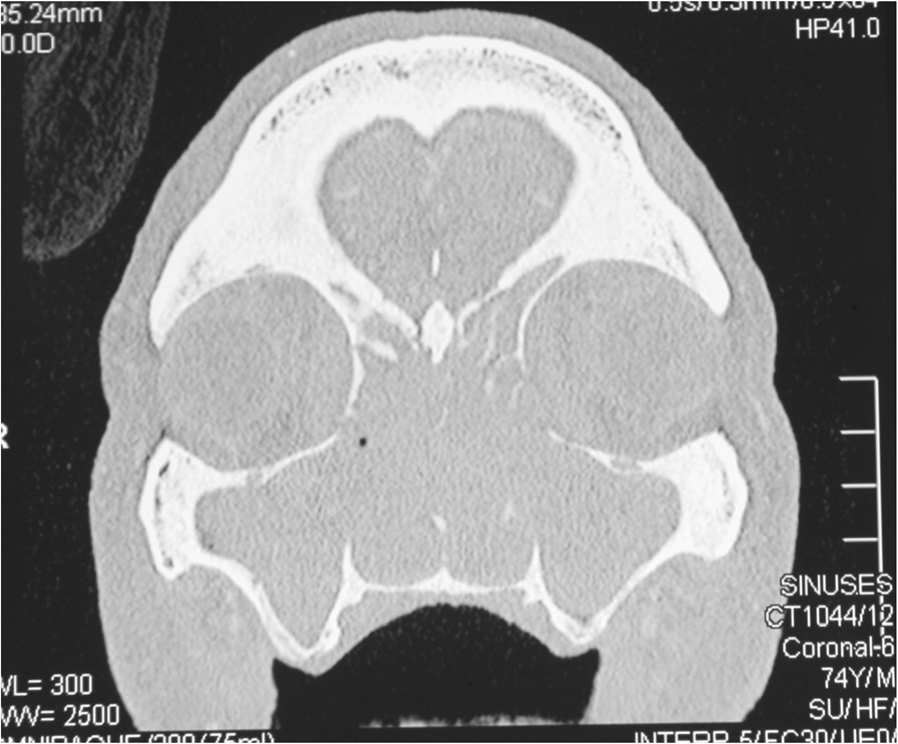
Coronal C.T: destruction of the medial walls of the maxillary sinuses and veiling of maxillary sinuses, ethmoidal sinus, frontal sinus and the nasal cavity.

**Figure 3:**
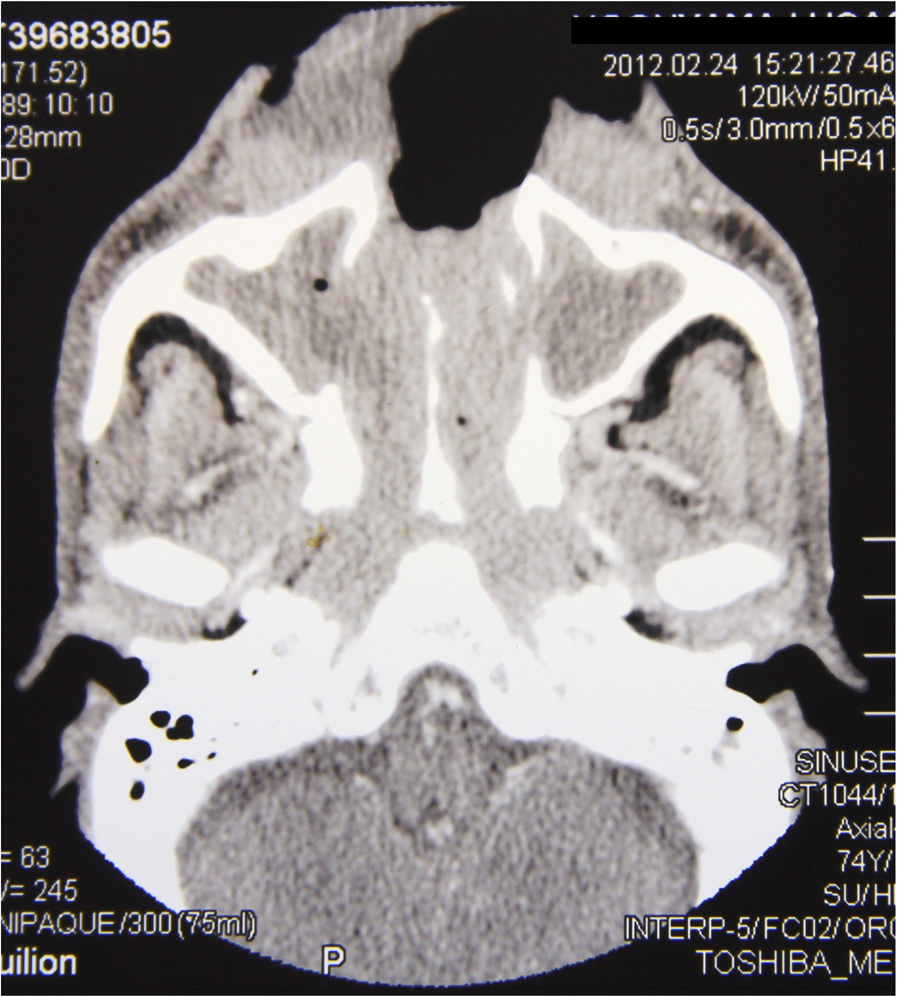
**Axial C.T: homogeneous soft tissue mass filling the anterior nasal cavity with bilateral obliteration of the maxillary sinuses, nasal cavity and the posterior nasal space.** Note the destruction of the anterior portion of the face, including the nose.

Computed tomographic (CT) scans showed radiopacity of a degree consistent with a soft tissue encroachment in the nasal cavity, both maxillary sinuses, ethmoidal, frontal and sphenoidal sinuses, and the nasopharynx (Figures 2 and 3).

**Figure 4:**
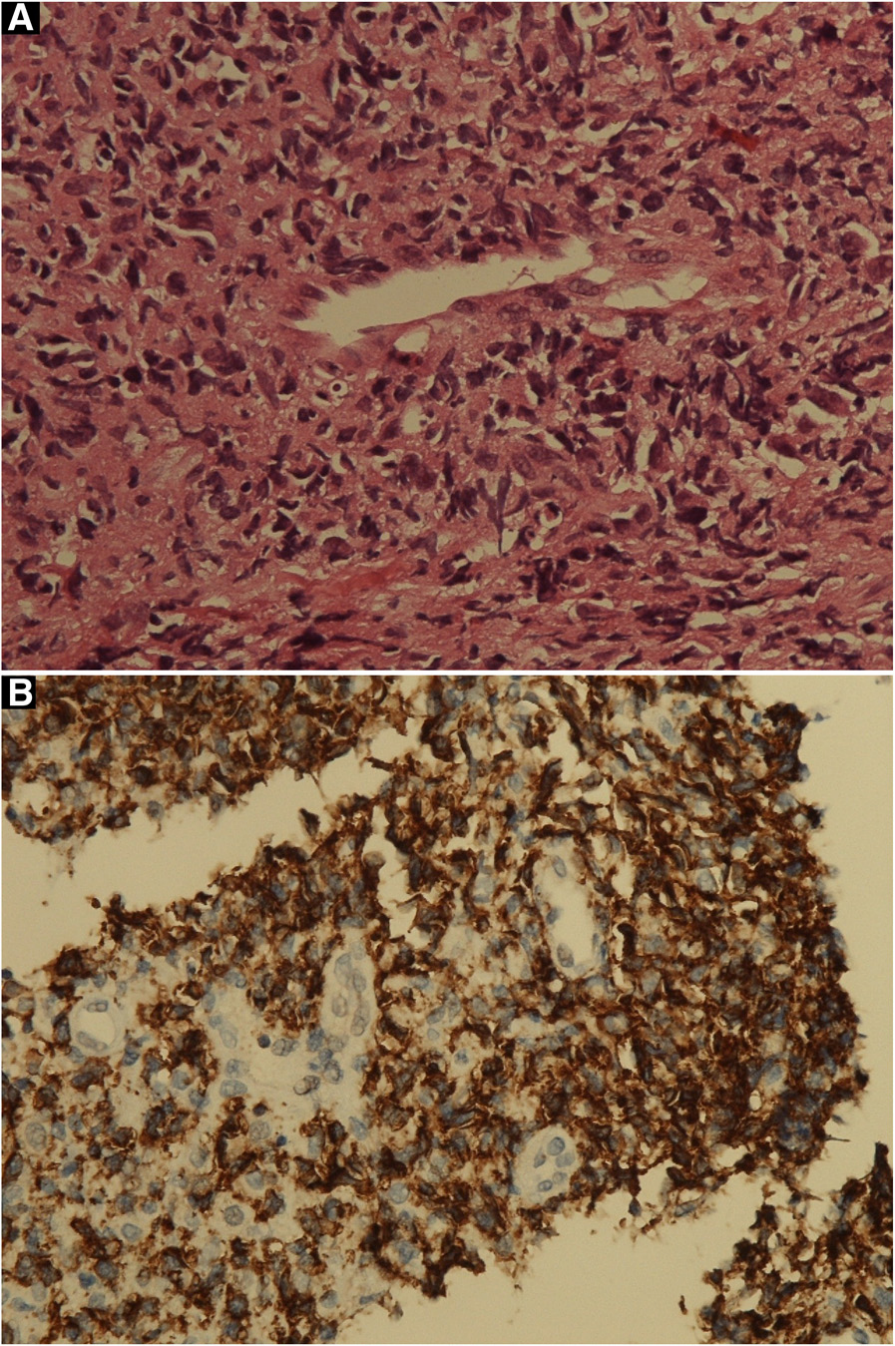
**NK/T cell lymphoma, nasal type. A**: atypical lymphocytes arranged in a vaguely angiocentric pattern (H&E, original magnification X 40). **B**: atypical lymphocytes positive for CD56 (original magnification X 40).

Biopsy of the tissue from the nasal mass showed a dense polymorphic cell infiltrate consisting of histiocytes, eosinophils and a great number of atypical lymphoid cells in an angiocentric distribution. The atypical lymphoid cells exhibited large nuclei, hyperchromatism, and some of them exhibited abnormal mitotic figures (Figure 4A). Areas of necrosis were evident.

Immunophenotypically, the tumour cells were positive for CD3 and CD56 (Figure 4B), but negative for CD20. Some of the large atypical lymphocytes were positive for CD30.

No facilities were available for *in situ* hybridization analysis which might have demonstrated whether the malignant cells harboured EBV small-encoded RNA.

As the clinical and histological features were consistent with a diagnosis of extranodal NK/T cell lymphoma, nasal type, the patient was referred to the oncology department for treatment, but he died soon after.

## Discussion

In the past, progressive destructive necrotic lesions involving the midface, the nose, paranasal sinuses and mouth, were referred to by the generic name ‘midline lethal granulomata’. However, with the advent of immuno-histochemical phenotyping methods it has become evident that ‘midline lethal granuloma’ comprises a heterogeneous group of disorders including non-Hodgkin lymphoma, Wegener granulomatosis and various granulomatous infections [2].

Extranodal NK/T cell lymphoma, nasal type, accounts for 7-10% of all non-Hodgkin lymphomas in Asia and Latin America, but for only 1% in Europe and North America [8,12,15]. In Korea, it accounts for about 75% of lymphomas arising within the nasal cavity and the paranasal sinuses [11]. To the knowledge of the authors, the prevalence of the disease in black South Africans, and indeed in black persons in general is unknown, but it appears to be very low.

Extranodal NK/T cell lymphoma, nasal type, is characterized histopathologically by angiocentric and angiodestructive growth, by tumour cells that vary in size and may harbour EBV in a clonal episomal form, and by an inflammatory cell infiltrate of plasma cells, histiocytes and eosinophils [1].

Immunophenotypically, the malignant tumour cells, like the natural killer cells from which they originate express CD2, cytoplasmic CD3 and CD56. In some cases they may also express cytotoxic granular-associated proteins, granzyme B, perforin, and T cell-restricted intracellular antigen (TIA-1). Genotypically, the malignant tumour cell expresses the T cell receptor gene in its germline configuration, but there is no monoclonal rearrangement of the T cell receptor [1]. However, there are cases in which the malignant cells lack CD56 and thus do not express the classic phenotype of NK cells; or they express an aberrant profile of CD8+ T cell antigens. Nevertheless these are well-recognised subsets of extranodal NK/T cell lymphoma, nasal type, because the clinical and histological features are characteristic [1].

As NK cells and T cells may arise from common progenitor cells, NK cells may express some T cell antigens, and T cells may express some NK cell antigens, so that cells of extranodal NK/T cell lymphoma, nasal type, may express both NK cell and T cell antigens [8].

Our patient’s tumour showed an angiocentric pattern of growth, and immunophenotypically, the tumour cells were CD56 positive, confirming the histological diagnosis of extranodal NK/T cell lymphoma, nasal type. Some extranodal NK/T cell lymphomas, nasal type consist of large cells expressing CD30 [1], and as the large atypical cells in our case were positive for CD30, the contention that our case is indeed extranodal NK/T cell lymphoma, nasal type, is supported.

NK cells express the cytoplasmic CD3 epsilon chain, but do not possess surface CD3 which is a T cell antigen. Since immuno-histochemical studies of paraffin sections with polyclonal anti-CD3 antibodies will disclose both cytoplasmic and surface CD3, this method cannot discriminate between NK cells and T cells. In order to distinguish between NK cells and T cells using cytoplasmic CD3 as a marker, immuno-histochemical studies must be performed on fresh-frozen tissue. Although, the immunohistochemical studies in our case were done on paraffin sections and not on fresh-frozen tissue, rendering the positive staining for CD3 inconclusive, nevertheless, this together with CD56 positivity, confirms an NK phenotype.

In the nose and paranasal sinuses, extranodal NK/T cell lymphoma, nasal type, is characterized by extensive local destruction of soft tissue, cartilage and bone, brought about by the aggressive angiocentric and angioinvasive nature of the tumour that results in functional incompetence of the vasculature with consequent ischaemia. This, together with the effect of cytotoxic molecules, including perforin, granzyme B and TIA-1 released by the malignant NK cells, and together with the tumour-associated inflammatory process, cause the widespread tissue damage and necrosis [4,6,16].

Typically, as seen on CT scans, the established soft tissue tumour obliterates the nasal passages and frequently invades and obliterates the maxillary sinuses [17]. Our patient presented with extensive facial destruction, and his extranodal NK/T cell lymphoma, nasal type, was diagnosed only late in the course of the disease, about one year after the initial symptoms. By then the tumour had already largely destroyed the structure of the nose and had invaded the maxillary, ethmoidal, frontal and sphenoidal sinuses, and the nasopharynx.

The extent of invasion by the tumour is classified by stages: T1 refers to a tumour confined to the nose; T2 to additional tumour invasion of the maxillary and anterior ethmoidal sinuses and/or the hard palate; T3 to further tumour invasion involving the posterior ethmoidal sinuses, sphenoidal sinuses, orbit, maxillary alveolar process of bone, and buccal tissues; and T4 to tumour invasion extending to the mandibular alveolar process of bone, to the infratemporal fossa, to the nasopharynx and to the cranial fossa [11,17]. Our patient was at the T3/T4 stage of the tumour.

The almost invariable presence of EBV in a latent clonal episomal form in the cells of the extranodal NK/T cell lymphoma, nasal type, strongly suggests a direct role of the virus in the pathogenesis of the tumour [1,6,18]. EBV infects the NK/T cells and establishes latent infection before initial transformation of NK/T cells has occurred, prior to the clonal divergence and clonal expansion of the cancerous cells. The presence of this clonotypic EBV genome in a latent form in the tumour cells strongly supports, but does not prove the pathogenic role of EBV in NK/T lymphomagenesis [7,19].

The active involvement of EBV in the pathogenesis of extranodal NK/T cell lymphoma, nasal type, is further supported by the direct positive correlation between EBV load in the tumour and the extent of the disease, and by the high titres of IgG antibodies to EBV in persons with the disease. Plasma titre of EBV DNA serves as a marker of tumour viral burden and fluctuates with the status of the disease and the response to treatment because EBV DNA fragments are released from apoptotic tumour cells and escape into the circulation [4,6,8].

At our hospital there are no facilities for *in-situ* hybridization studies, so we do not know whether or not the cells of the extranodal NK/T cell lymphoma, nasal type, of our patient carried EBV encoded early RNA, but serological studies showed that our patient had IgG but not IgM, to EBV cuspid antigen, indicative of either past EBV infection, or of current latent infection.

## Conclusion

We present a case of extranodal NK/T cell lymphoma, nasal type in an elderly black male. This tumour appears to be rare in black persons, as we could find only a few cases reported in the literature. It may well be that the incidence is higher, but because cases in remote rural communities in sub-Saharan Africa are unlikely to be reported, the epidemiology of the tumour in this geographic area is unknown.

The purpose of this article is to add to the information available with regard to extranodal NK/T cell lymphoma, nasal type in black persons in the hope that data gathered from single case reports will help to characterise this disease in black persons, and to help identify the biological/environmental factors which confer protection against developing the disease in this population group.

## Consent

We declare that the patient had given consent for the case report to be published.

## Competing interests

The authors declare that they have no competing interests.

## Authors’ contribution

MMT and MK treated the patient and collected the data. MB performed histopathological examination and immunohistochemical staining. RAGK, MB, JL and LF analysed and interpreted the data. The concept of the paper was devised by LF. RAGK, JL and LF wrote the manuscript. All authors read and approved the final manuscript.
